# Limits on using the clock drawing test as a measure to evaluate patients with neurological disorders

**DOI:** 10.1186/s12883-022-03035-z

**Published:** 2022-12-31

**Authors:** Raheleh Heyrani, Atiye Sarabi-Jamab, Jordan Grafman, Nesa Asadi, Sarvenaz Soltani, Fatemeh Sadat Mirfazeli, Mostafa Almasi-Dooghaei, Seyed Vahid Shariat, Amin Jahanbakhshi, Tara Khoeini, Mohammad Taghi Joghataei

**Affiliations:** 1 Department of Psychiatry, School of Medicine, Mental Health Research Center, Psychosocial Health Research Institute, Iran University of Medical Sciences, Tehran, Iran; 2 School of Cognitive Sciences, Institute for Research in Fundamental Sciences (IPM), Tehran, Iran; 3 Shirly Ryan AbilityLab, Departments of Physical Medicine and Rehabilitation, Neurology, Cognitive Neurology, and Alzheimer’s Center, Chicago, IL USA; 4 Department of Psychiatry, Feinberg School of Medicine and Department of Psychology, Weinberg College of Arts and Sciences, Northwestern University, Chicago, IL USA; 5 Faculty of Medicine, Rasool Akram Hospital, Iran Unversity of Medical Sciences, Tehran, Iran; 6 Department of Neurology, Firoozgar Hospital, School of Medicine, Iran University of Medical Sciences, Tehran, Iran; 7 Department of Neurosurgery, Skull Base Research Center, Rasool Akram Hospital, Iran University of Medical Sciences, Tehran, Iran; 8 Stem Cell and Regenerative Medicine Research Center, Iran University of Medical Sciences, Tehran, Iran; 9 Cellular and Molecular Research Center (CMRC), Iran University of Medical Sciences, Tehran, Iran

**Keywords:** The clock drawing test, Diagnosis, Screening tool, Location of brain lesions

## Abstract

**Background:**

The Clock Drawing Test (CDT) is used as a quick-to-conduct test for the diagnosis of dementia and a screening tool for cognitive impairments in neurological disorders. However, the association between the pattern of CDT impairments and the location of brain lesions has been controversial. We examined whether there is an association between the CDT scores and the location of brain lesions using the two available scoring systems.

**Method:**

One hundred five patients with brain lesions identified by CT scanning were recruited for this study. The Montreal Cognitive Assessment (MoCA) battery including the CDT were administered to all partcipants. To score the CDT, we used a qualitative scoring system devised by Rouleau et al. (1992). For the quantitative scoring system, we adapted the algorithm method used by Mendes-Santos et al. (2015) based on an earlier study by Sunderland et al. (1989). For analyses, a machine learning algorithm was used.

**Results:**

Remarkably, 30% of the patients were not detected by the CDT. Quantitative and qualitative errors were categorized into different clusters. The classification algorithm did not differentiate the patients with traumatic brain injury ‘TBI’ from non-TBI, or the laterality of the lesion. In addition, the classification accuracy for identifying patients with specific lobe lesions was low, except for the parietal lobe with an accuracy of 63%.

**Conclusion:**

The CDT is not an accurate tool for detecting focal brain lesions. While the CDT still is beneficial for use with patients suspected of having a neurodegenerative disorder, it should be cautiously used with patients with focal neurological disorders.

**Supplementary Information:**

The online version contains supplementary material available at 10.1186/s12883-022-03035-z.

## Introduction

When evaluating patients with neurological disorders, finding a test that is easy and quick to administer is helpful in clinical practice. One such test is the Clock Drawing Test (CDT). Using clock drawings to test patients was first described by the British neurologist Sir Henry Head [1]. It has been used more often since the 1960s and it became especially popular when it was added to the Boston Aphasia Battery by Goodglass and Kaplan in 1983.

The CDT was originally used for diagnostic purposes to screen for dementia [2–6]. Currently, its use has expanded to screen for cognitive impairments in other neurological disorders including hypertension-mediated brain damage [7], focal brain damage in patients with traumatic brain injury (TBI) [8, 9], and stroke [10–13]. In a retrospective study with TBI patients, results with the CDT demonstrated that patients with subarachnoid hemorrhage, brain edema, parietal, and bilateral injuries had lower scores than patients without a subarachnoid hemorrhage [14].

However, the association between specific errors on the CDT and the location of brain lesions has been controversial [10, 14, 15]. Even though CDT has been associated with parietal lobe dysfunction [14], many studies have found that CDT performance is linked to several brain regions including the left and the right posterior and middle temporal lobe, the right middle frontal gyrus, and the right occipital lobes [16, 17]. Involvement of the parietal-temporal and frontoparietal cortical networks in healthy individuals’ CDT performance has been shown by an fMRI study [16]. In another fMRI study, increased activation was observed in the bilateral frontal, occipital and parietal lobes, supplementary motor area, and pre-central gyrus during the administration of the CDT in healthy aged people [18]. In sum, it seems that CDT employs several different areas of the brain, and its activated regions are not limited to only a single of two isolated areas. This wide activation profile undermines the possibility that the CDT might have some potential to identify the underlying injuries in brain lesion patients.

Complicating this effort is that several different qualitative and quantitative scoring systems have been used to analyze CDT errors [14, 16–21]. One of the most commonly used qualitative scoring systems is one advocated by Rouleau and colleagues [22]. On the other hand, quantitative CDT scoring systems like those of Shulman (2000) [23] and Sunderland et al. (1989) [24] have also been advocated. A qualitative analysis of the CDT was able to predict the progression of dementia in non-demented older adults [25]. In that particular study, a regression analysis showed the existence of CDT conceptual deficits that were significantly associated with the progression to dementia 1 year after the initial assessment of cognitive function. On the other hand, Dong et al. (2020) [26] concluded in their study that a combination of the CDT quantitative scores with qualitative observations of the clock-drawing errors provided better discrimination between vascular MCI patients and cognitively normal subjects. However there is a paucity of research using both CDT quantitative and qualitative scoring systems to determine whether the combined scoring systems would be helpful in discriminating between types of neurological disorders or the location of lesions and to our knowledge, none of them have used a machine learning approach.

Machine learning is a powerful tool that has been successfully used in medicine to help in diagnosis [27–31]. This is especially useful when there are complicated scoring systems and no predetermined and distinct differentiating criteria exist to classify the subgroups of patients [32, 33]. We hypothesized that combining the two scoring systems of CDT with a powerful machine learning method could more accurately test the ability of CDT in localizing brain lesions.

Finally, the CDT is still in use in many countries as a screening measure because it is easy to administer, feasible for individuals with severe brain pathology to complete, and can be completed quickly [2–10, 12, 13]. Therefore, knowing about its advantages and limitations could be very helpful in clinical decision-making.

Therefore, in this study, we aimed to see what extra data will be provided by the CDT, analyzing patients with cognitive impairment. We evaluated the validity of two popular CDT scoring systems to see whether the CDT scoring systems could detect brain lesions and provide information regarding cognitive impairment in patients without progressive neurodegenerative disease. We then used machine learning algorithms to detect the different patterns and features of CDT performance that could help to classify the location of brain lesions.

## Methods

### Participants

One hundred five patients who were referred to the neuropsychiatry or neurosurgery clinics of three referral hospitals of the Iran University of Medical Sciences between 2018 and 2021 agreed to participate in this study. They were aged between 21 and 77-years-old, had a variety of acquired brain lesions due to stroke, traumatic brain injury (mostly closed injury), brain tumor, and brain aneurysm surgery. Patients who were in the intensive care unit and medically unstable as well as severely confused and agitated patients were excluded. To participate, patients were required to have at least a fifth-grade education with illiterate patients excluded.

### Demographic measurements

Measures of age, gender, marital status, education, occupation, surgical intervention, the existence of epilepsy, and GCS were obtained (see Table 2).

### Cognitive assessment

The cognitive abilities of patients were assessed by the Montreal Cognitive Test (MoCA). This test was designed by Nasreddine et al. in 2005 [34] to detect mild cognitive impairment (MCI). It contains seven domains of cognitive functioning including visuospatial executive functions, naming, attention, language, abstraction, delayed recall, and orientation and contains a CDT. For the CDT subtest of the MoCA, each patient was given a white A4 paper and was asked to draw the clock and set the time to 10 minutes after 11 o’clock.

### Scoring systems

Each clock was scored with the scoring checked by two neuropsychologists and one neuropsychiatrist. We used two types of scoring: one qualitative and one quantitative. For the qualitative scoring system, we used the Rouleau procedure [22] in which five kinds of errors can be categorized: error 1) graphical difficulties: when the lines are not precise, the clock face is distorted and the numbers cannot be read; error 2) stimulus-bound response: when the participant focuses on one single stimulus often related to time-setting. For example, the time 11:10 has to be set but the patient incorrectly places the clock hands, error 3) conceptual deficits: when there is misinterpretation of the features or meaning of the clock, error 4) spatial and/or planning deficits: when errors occur in drawing the layout of the clock, for example, the space between the numbers or neglect of one side of the clock and error 5) perseveration: when the continuation of the requested features in clock recur, for example, drawing more than two hands, an ongoing trace of the clock face line or preserved numbers [35].

For the quantitative scoring system, we used the procedure advocated by Mendes-Santos et al. (2015) [36] based on the Sunderland et al. (1989) study [24]. The sensitivity and specificity of the CDT using the Sunderland system are72.6 and 87.9% as reported in a systematic review [37] (See Table 1).

**Table 1:** Criteria for scoring the CDT using Mendez-Santos et al. (2015) scoring system

a) Presence of a circle b) Presence of the 12 numbers c) Numbers entered within the internal part of the clock d) Numbers entered in the correct ascending order. e) Numbers entered in the correct spatial margin f) Ability to draw a vertical straight line between 12 and 6 g) Ability to draw a horizontal straight line between 3 and 9 h) Numbers are not focused on one side of the clock i) Presence of two pointers j) Presence of the hour hand k) Presence of the minute hand l) The minute hand is longer than the hour hand m) One of the hands has to be between 2 and 3 to show the minute n) One of the hands has to be between 10 and 11 to show the hour o) Wrong use of hands whether drawn digital type or circling the numbers inappropriately p) Some clues show that the requested task is understood as a clock. q) It did not represent a clock or the patient did not try to draw a clock.	
Additional scoring included the following items: 1. If the item “o” is checked, the score is will be 6 points. 2. If the item “p” is checked, the score will be 2 points. 3. If the item “q” is checked, the score will be 1 point.	
If the clock and the numbers are drawn correctly, the score will be between 6 and 10 and if they are drawn incorrectly, the score will be between 1 and 5:^a^ 1. The patient did not try or did not represent a clock (presence of “X” in item q) 2. Only a little evidence exists that shows an understanding of the clock (“X” in p) 3. No hands (No “X” in I, j, k) 4. Missing Numbers internally and located outside of the clock (No “X” in b and c) 5. Reversed order of the numbers of concentrated shapes (no “X” in d or h) 6. Wrong use of hands (presence of “X” in item o) 7. Severe deficit of the hands (No “X” in the items of l, m, n) 8. Mild deficit of the hands (No “X” in at least 2 items of l, m, n) 9. Very mild deficit of the hands (absence of “X” in at least one item: l, m or n) 10. Correct time (no “X” in the items: o, p, q)	

^a^According to the Mendez-Santos et al. (2015) method, the examiner has to mark with an “X” if the following items are present

### Imaging report

We used CT scans to detect the location of brain lesions. The CT scan images were obtained close to the date the patients received their neuropsychological assessment. Consensus about the location and extent of the brain lesion was reached by two neurosurgeons and neurologists.

### Analysis

Our interpretation of the results was guided by Eknoyan et al. (2012) [35] who suggested that different kinds of CDT errors indicate the location of a damaged brain area. They demonstrated that based on several studies, error 1, graphic difficulties, are the result of a secondary disruption of frontostriatal circuits-necessary for coordinating fine motor control and planning; error 2, a stimulus-bound response are the result of frontostriatal circuits impairment leading to executive-function deficits; error 3, conceptual deficits, are the result of brain injuries in the left inferior frontal-parietal opercular cortices which are associated with time setting errors or are likely due to an impairment in semantic memory which is a primary function of the lateral temporal lobes; error 4, spatial and/or planning deficits, which could be the result of deficits in frontoparietal circuits- and play an important role in coordinating the visuospatial understanding of a clock and the result of frontostriatal circuits which are responsible for aspects of executive function for an accurate clock face; and error 5, preservation, are the result of impairment of executive function in the prefrontal cortex.

Given the results reported in Eknoyan et al. (2012) [35], we focused our analyses on the frontal, temporal, parietal, and subcortical brain regions.

### Statistical analysis

All statistical analyses were performed using R version 3.6.1 (R Core Team, 2019) along with R Studio and the “dplyr” and “rlang” packages for data manipulation. We utilized the “ggplot2” for data visualization. For numerical variables, we used the mean and standard deviation (SD) when they were normally distributed, and the median and range if they were not. We used analysis of variance (ANOVA) to compare differences between category mean scores. The correlation was tested using Pearson’s correlation coefficient.

To find the predictor factor in our dependent variables, we performed a (generalized) linear model regression analysis, and in the case of more than one predictor factor, we used the “MASS” package which chooses the best model based on the Akaike information criterion (AIC). We also used the “stepAIC” function for stepwise regression and the “rsq” package to calculate the R-squared and partial correlation coefficients for generalized linear (mixed) models. To support multi-label classification processing, we used the “utiml” package, which it provides a set of multi-label procedures such as sampling methods, transformation strategies, threshold functions, pre-processing techniques and evaluation metrics. Statistical significance was set at a 2-tailed *p*-value threshold of < 0.05.

## Results

Demographic information of the patients is presented in Table 2.

**Table 2:** Demographic information of 105 patients with brain lesion

Characteristics	105 Patients
Age, mean (SD)	39.25 (14.95)
Sex, N	105
Female	16
Male	89
Marital Status, N	105
Single	28
Married	68
Unknown	9
Education, N	105
Undergraduate	94
Graduate	9
Unknown	2
Injury, N	105
TBI	75
Non-TBI	30
Type of lesion	105
TBI	75
Brain tumor	7
Stroke	13
Aneurysm	2
Hydrocephaly	1
Status epileptic	2
IVH	1
Normal CT	4
Passed months since injury	
Minimum	1
Maximum	48
Mean (SD)	7.33 (11.49)
Surgery, N	105
History of surgery	56
No surgery	49
Epilepsy, N	105
Epileptic	11
No epilepsy	87
Unknown	7

The mean and standard deviation (SD) of the CDT score using the Mendes-Santos et al. (2015) Scoring procedures was 7.52 (2.82).

We then tried to categorize a large number of qualitative and quantitative scoring variables using a hierarchical clustering approach. This resulted in an attractive tree-based dendrogram representation of the observations. In this approach, each score is initially considered as a single-element cluster, and at each step of the algorithm, the two clusters that are the most similar are combined into a new bigger cluster. This procedure is iterated until the dendrogram tree is completely generated. Power dissimilarity is used to calculate the distance between two entities whose attribute has categorical values. The dissimilarity between two clusters is calculated based on minimizing the total within-cluster variance.

**Fig. 1:**
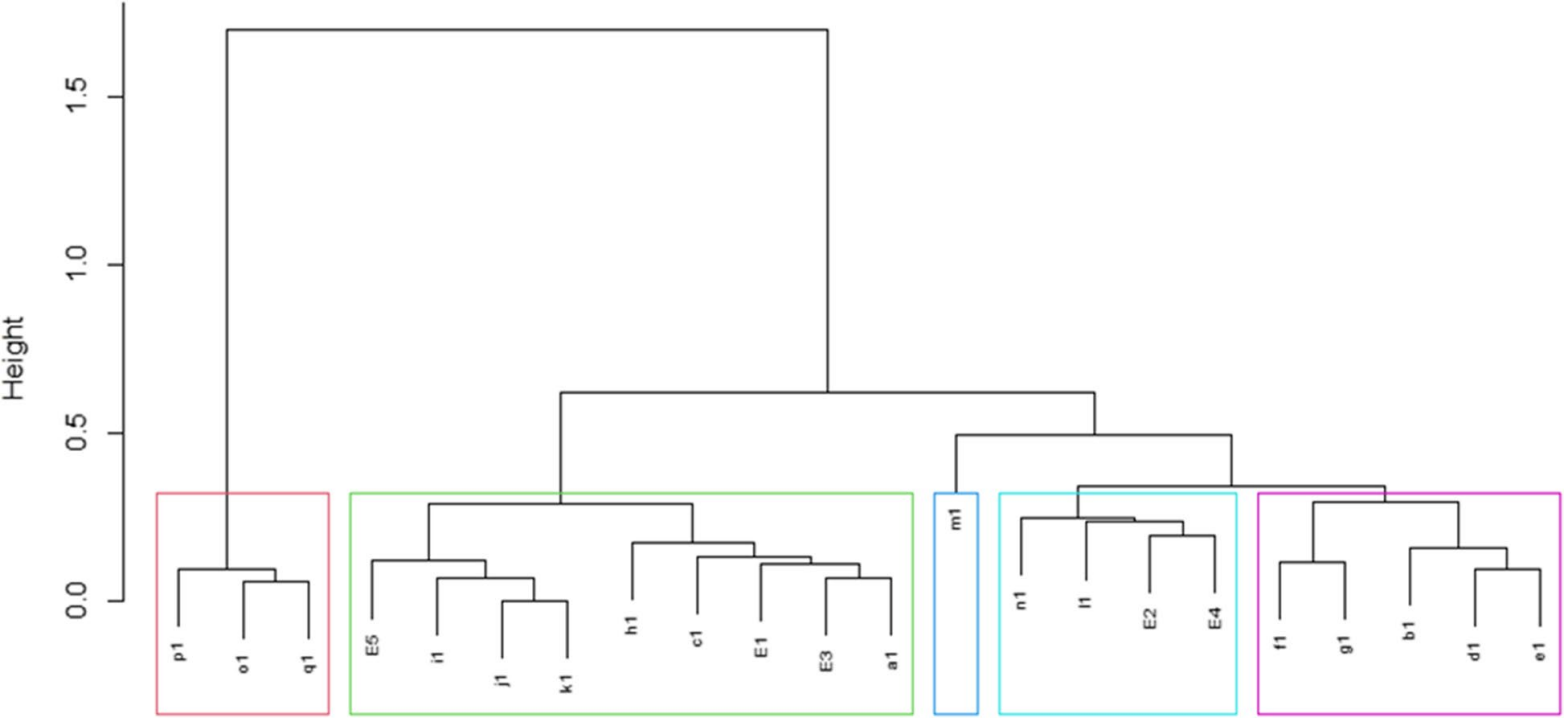
Dendrogram showing hierarchical analysis of both qualitative and quantitative measures of CDT

**Fig. 2:**
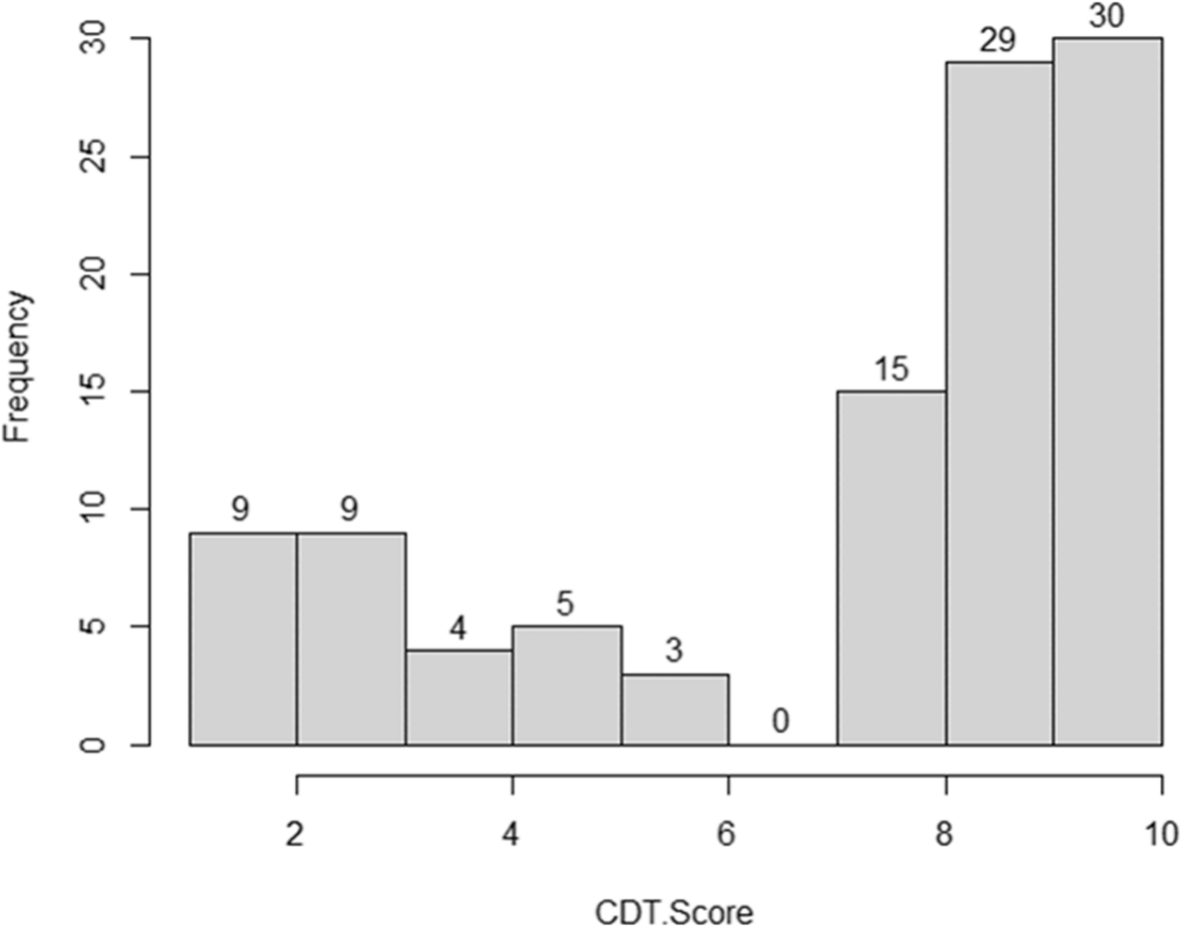
Performance of CDT in 105 patients with brain lesion

In Fig. 1, E1 to E5 indicate graphical difficulties, stimulus-bound response, conceptual deficit, spatial and/or planning deficits, and perseveration errors, respectively. Moreover, a1 to q1 indicate quantitative error basis vectors based on the Mendes-Santos, et al. scoring system. As shown in Fig. 2, p_1_, o_1,_ and q_1_ are in the first cluster on the left side of the dendrogram in pink; E_5_, i_1_, j_1,_ and k_1_ from another cluster which is near a second cluster including h_1_, c_1_, E_1_, E_3_ and a_1_ and these two clusters form one bigger cluster in green; m_1_ is a single cluster by itself in dark blue; n_1_, l_1_, E_2_ and E_4_ form another light blue cluster and f_1_, g_1_, b_1_, d_1_, and e_1_ are contained in the final purple cluster, respectively. The pink cluster seems to refer to the understanding of the whole generality of the clock concept. The green cluster might be likely due to impairment of executive function [38, 39], associated with time-setting errors [21], or could also be due to impairments in semantic memory [3]. The dark blue cluster has the same function as the previous one. The light blue cluster appears to be related to time-setting instructions [35] and the inability to coordinate the visuospatial understanding of a clock [21]. Finally, the purple cluster might be considered the visuospatial and executive function [35].

The histogram shown in Fig. 2 shows the patients’ performance on the CDT as characterized by the Mendes-Santos et al. Scoring method. The frequency of a perfect score of “10″ was 30%. The frequency of the nearly perfect score “9″ in the CDT was 29%. Scores “1″ and “7″ are not shown in the current figure because patients in our study did not receive those scores.

**Fig. 3:**
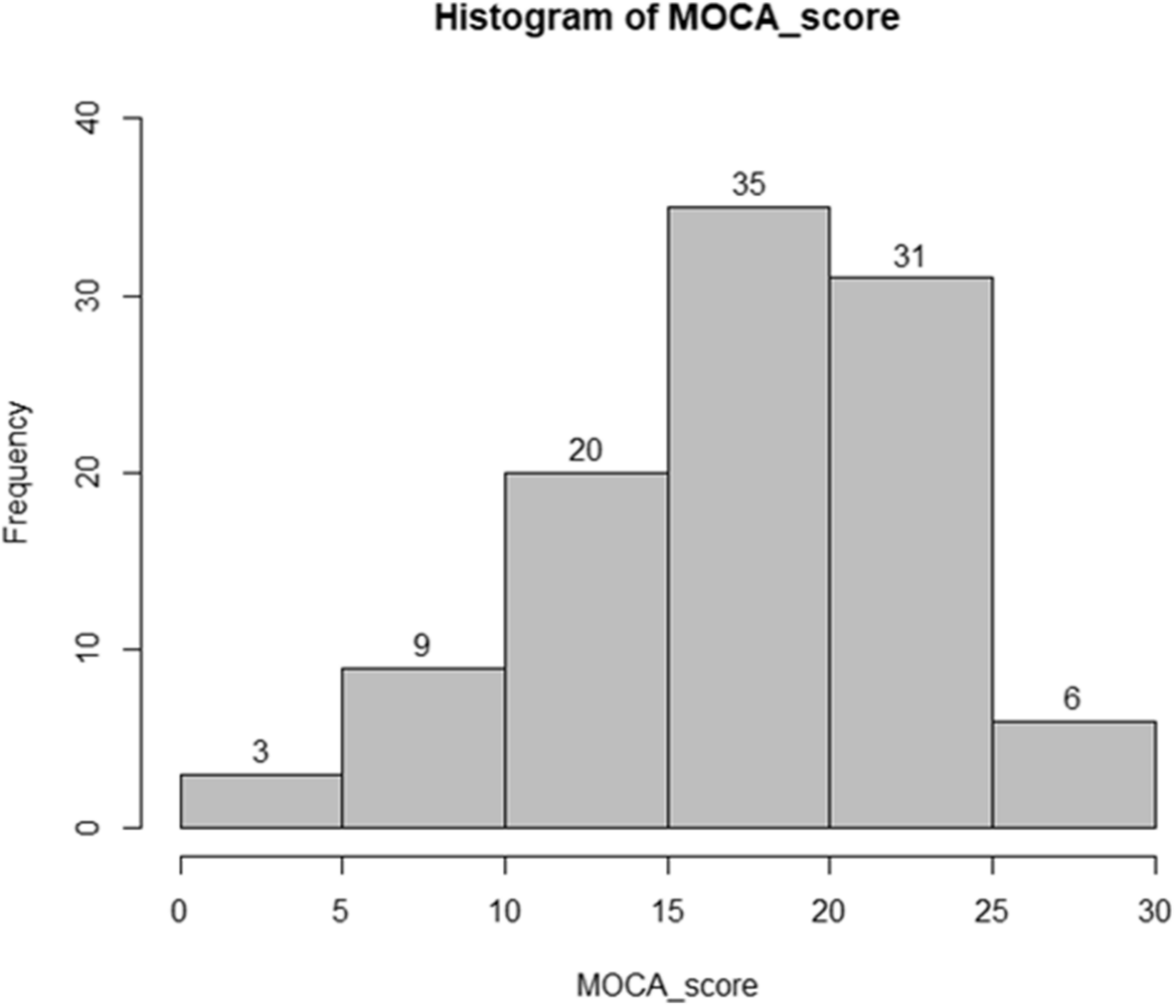
Distribution of MoCA scores in 105 patients with brain lesions

The histogram shown in Fig. 3 shows the patients’ performance on the MoCA. The frequency of scores between ‘15–20′ was 35% which was the highest rank, scores between ‘20–25′ was 31% and scores between ‘10–15′ was 20%.

As Table 3 shows, we checked if the history of surgery as well as the history of epilepsy could change the performance of CDT. The results of the regression model are as follows (See Table 3):

**Table 3:** Regression model regarding history of surgery, and epilepsy to change the CDT performance in 105 patients with brain lesions

Coefficients	Estimate Std	Error	T value	Pr
(Intercept)	9.94	2.90	3.42	.00 ***
Surgical interventions	.05	.60	0.01	.92
Epilepsy (no)	−2.40	2.87	−.83	.40
Epilepsy (yes)	−2.44	2.97	−.82	.41

In our study, we just had the record of the past months since injury for 35 participants, and for these patients, we checked if the passed months since the injury, the history of surgery as well as the history of epilepsy could change the performance of CDT. The results of the regression model are shown in Table 4:

**Table 4:** Regression model regarding history of surgery, and epilepsy to change the CDT performance in 35 patients with clear history of the past months since injury

Coefficients	Estimate Std	Error	T value	Pr
(Intercept)	6.60	.93	7.06	6.15e ***
Surgical interventions (yes)	.05	.60	0.01	.92
Epilepsy (yes)	−2.40	2.87	−.83	.40
Passed months injury	−2.44	2.97	−.82	.41

Thirty patients out of 105, achieved a perfect score of 10 (see Supplementary Material, Table 1). Out of these 30 patients, 27% had brain lesions in the frontal lobe (C1), 27% patients had brain lesions in the temporal lobe (C2), 7% had brain lesions in the parietal lobe (C3), 3% had brain lesion in a subcortical area, 17% had brain lesions in the frontal lobe and temporal lobe (C1, C2), 10% had brain lesions in the temporal lobe and parietal lobe (C2, C3), 3% had brain lesions in the frontal lobe and parietal lobe (C1, C3), and 3% had brain lesions in the frontal, temporal and parietal lobes (C1, C2, C3). Thus, more than half of the patients with an intact test result despite a CT scan verified brain lesion, had lesions in frontal or temporal lobes that were considered critical for the performance of the CDT in previous studies [16–18].

We next used traditional machine learning methods to try and build predictive models for further analysis. First of all, we examined whether we could discriminate among the neurological disorders in our patients and determined whether any of the domains of the MoCA along with CDT could predict the existence of a TBI or not. Therefore, we built a logistic regression model including all of the features from the MoCA and a few demographic variables and compared this full model to a series of models omitting each variable in turn.

As it is shown in Table 5, Age was the only predictor that linearly correlated with the type of injury (t = − 3.22, *p* < .05) and could differentiate between TBI and non-TBI patients.

**Table 5:** Logistic regression model to assess the relationship between each features of MoCA or CDT score and the type of injury (TBI/Non-TBI)

Intercept	Estimate	Standard Error	t value	Sig
CDT score	−.009	.04	−.25	.804
MoCA	−.067	.11	−.60	.547
MoCA-without CDT	.034	.10	.34	.734
Visuospatial Executive Function in MOCA test	.039	.08	.49	.625
Naming	.025	.11	.22	.823
Attention	.087	.05	1.56	.121
Language	.007	.06	.10	.91
Abstraction	.044	.07	.56	.57
Delayed Recall	.050	.05	.89	.387
Orientation	−.018	.04	−.37	.707
Age	**−.011**	**.003**	**−3.22**	.001**
Sex	.127	.14	.87	.386

*P* < .05 is significance level **

We next adopted a nonlinear approach for classification using the support vector machine (SVM) procedure. SVM is a generalization of a maximal margin classifier, in which the underlying goal is to draw a hyper-plane through a set of observations that separates the data into two classes. We examined if the CDT score, in particular, could be a good predictor of the type of injury.

As is demonstrated in Table 6, after the exclusion of the Age variable, our classification algorithm could differentiate TBI from non-TBI patients with an accuracy of 74% and the detection of true positives in non-TBI patients was very low.

**Table 6:** Nonlinear classification using SVM method to check if the CDT score could predict the type of injury

	TP	FP	FN	TN	Correct	Wrong	TP %	FP %	FN %	TN %	Correct %	Wrong %	Mean Ranking	Mean Score
TBI	24	7	2	1	25	9	.71	.21	.06	.03	.74	.26	1.09	.8
Non-TBI	1	2	7	24	25	9	.03	.06	.21	.71	.74	.26	1.91	.2

Additionally, we checked if we could predict the hemispheric location of injury using the same classification algorithm.

As seen in Table 7, after exclusion of Age, our classification algorithm was only able to predict the hemisphere of injury with an accuracy of 58% and the rate of true positives for the detection of brain lesions in the left hemisphere was very low.

**Table 7:** Nonlinear classification using SVM method to check if the CDT score could predict the location of injury in the Left lobe/ Right Lobe

	TP	FP	FN	TN	Correct	Wrong	TP %	FP %	FN %	TN %	Correct %	Wrong %	Mean Ranking	Mean Score
Left Lobe	0	1	7	11	11	8	.00	.05	.37	.58	**.58**	.42	1.95	.19
Right Lobe	11	7	1	0	11	8	.58	.37	.05	.00	**.58**	.42	1.05	.78

As it can be seen in Table 8, only attention is the predictor that has the effect (t = 2.45, *p* < .05).

**Table 8:** Logistic regression model to control for the effects of predictors on the “left vs. right injury” status

Intercept	Estimate	Standard Error	t value	Sig
CDT score	−.033	.041	−.81	.41
MoCA	−.277	.16	−1.77	.08
MoCA-without CDT	.150	.14	1.09	.27
Visuospatial Executive Function	.039	.08	.49	.06
Naming	.091	.14	.63	.52
Attention	**.189**	**.07**	**2.45**	.01*
Language	.026	.10	.25	.80
Abstraction	.215	.11	1.85	.07
Delayed Recall	.165	.08	2.00	.05
Orientation	.046	.06	.67	.50
Age	−.008	.006	−1.38	.17
Sex	−.148	.21	−.70	.50
Injury type	−.227	.17	−1.30	.20

*P* < .05 is significance level *

We used machine learning algorithms in an attempt to classify different areas of brain injury using qualitative and quantitative error features and patterns of performance (the class labels are y1: Frontal, y2: Temporal, y3: Parietal, y4: Sub-cortex).

As seen in Table 9, the classification algorithm used the MoCA test without the clock measure subscale and the combined qualitative and quantitative scoring of the CDT and it was poor in predicting lesion location. Predicting a frontal lobe lesion only achieved an accuracy of 42%, predicting a temporal lobe lesion achieved an accuracy of 42%, predicting a parietal lobe lesion achieved an accuracy of 63% but predicting a subcortical lesion achieved a high accuracy of 95%. However, this high level of accuracy is probably due to the very small sample size of patients with subcortical brain lesions.

**Table 9:** Nonlinear classification using SVM method to check if the CDT score could predict the location of brain injury

	TP	FP	FN	TN	Correct	Wrong	TP %	FP %	FN %	TN %	Correct %	Mean Ranking	Mean Score
Y1 Frontal lobe	8	7	4	0	8	11	.42	.37	.21	.00	**.42**	1.58	.63
Y2 Temporal lobe	2	3	8	6	8	11	.11	.16	.42	.32	**.42**	2.05	.41
Y3 Parietal lobe	2	3	4	10	12	7	.11	.16	.21	.53	**.63**	2.37	.32
Y4 Sub-cortex	0	0	1	18	18	1	.00	.00	.05	.95	**.95**	4.00	.06

Furthermore, to examine the sensitivity and specificity of quantitative CDT scoring system, qualitative CDT scoring system, and combined systems in the patients with cognitive impairment (identified with MoCA) we divided the patients into two groups: patients with cognitive impairments (MoCA< 26), and patients without cognitive impairments (MoCA> = 26).

For the qualitative CDT score, if there is any error, it detects the patients. Moreover, for the quantitative CDT score, it is an impairment, if the score is less than 9.

Finally, we could measure sensitivity, and specificity as follows:

- sensitivity = TP/(TP + FN).
- specificity = TN/(TN + FP).

**Fig. 4:**
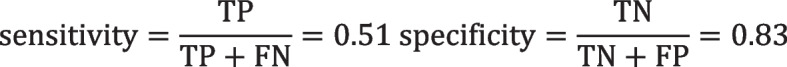
The sensitivity and specificity of the CDT in cognitive impaired patients based on the qualitative CDT scoring

**Fig. 5:**
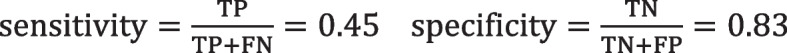
The sensitivity and specificity of the CDT in cognitive impaired patients based on the quantitative CDT scoring

**Fig. 6:**
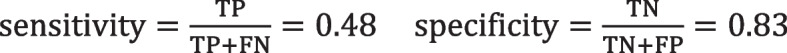
The sensitivity and specificity of the CDT in cognitive impaited patients based on the combination of both qualitative and quantitative scoring systems

As you could see the sensitivity and specificity of the qualitative and quantitative and combined scoring systems in Figs. 4,5, and 6 respectively, the sensitivity of all systems are not high and they all show fairly good specificity. (See Figs. 4, 5 and 6).

One possible way to make this study more informative is by analyzing patients with cognitive impairment (identified by MoCA), and ascertaining whether the CDT could be useful in detecting it. So, we evaluated the Pearson correlation coefficients between the CDT score and each of the MoCA subscales.

**Fig. 7:**
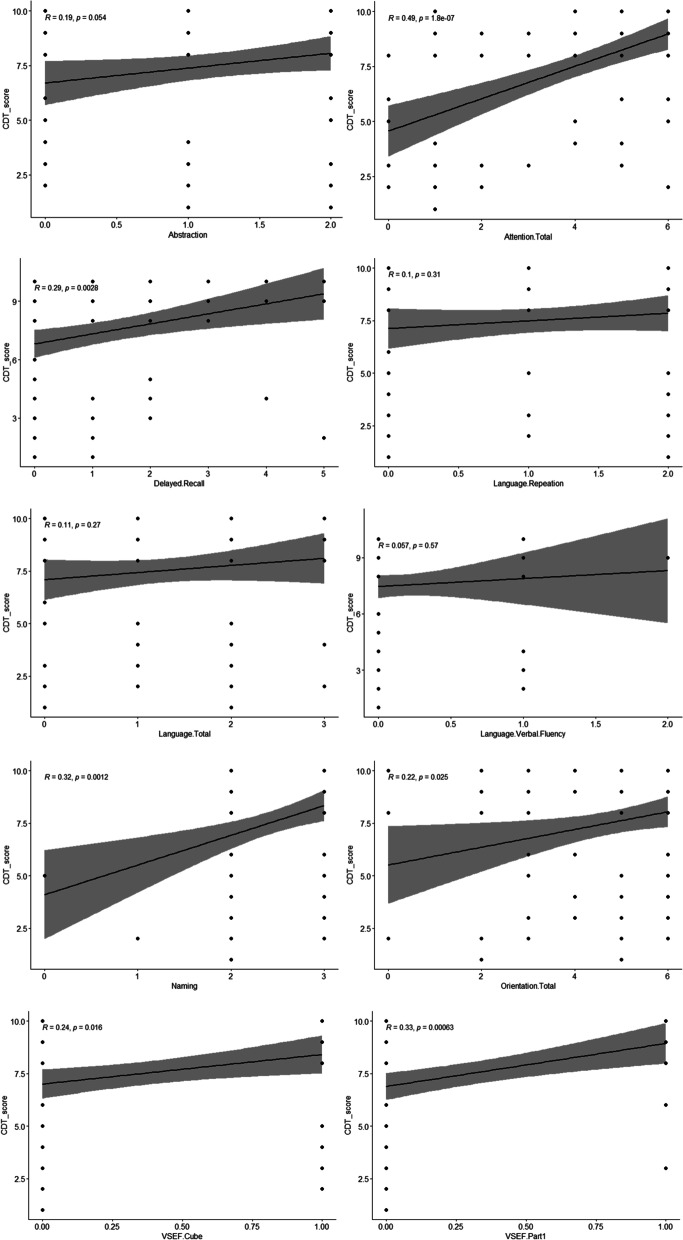
First series of the Correlation coefficient between the CDT and the subscales of the MoCA (full CDT scores)

As it is shown in Fig. 7, the MoCA subscales including attention total (*r* = .49, *p* = 1.8), language repeatition (*r* = .01, *p* = .31), language verbal fluency (*r* = .057, *p* = .57), language total (*r* = .11, *p* = .27), and abstraction (*r* = .19, *p* = .05) were not correlated with the CDT. There was a very low positive correlation between the CDT and the other sucbscales: delayed recall (*r* = .29, *p* = .002), naming (*r* = .32, *p* = .001), orientation (*r* = .22, *p* = .22), visual spatial executive function cube (*r* = .24, *p* = .0016), visual spatial executive function series part one (*r* = .33, *p* = .000).

**Fig. 8:**
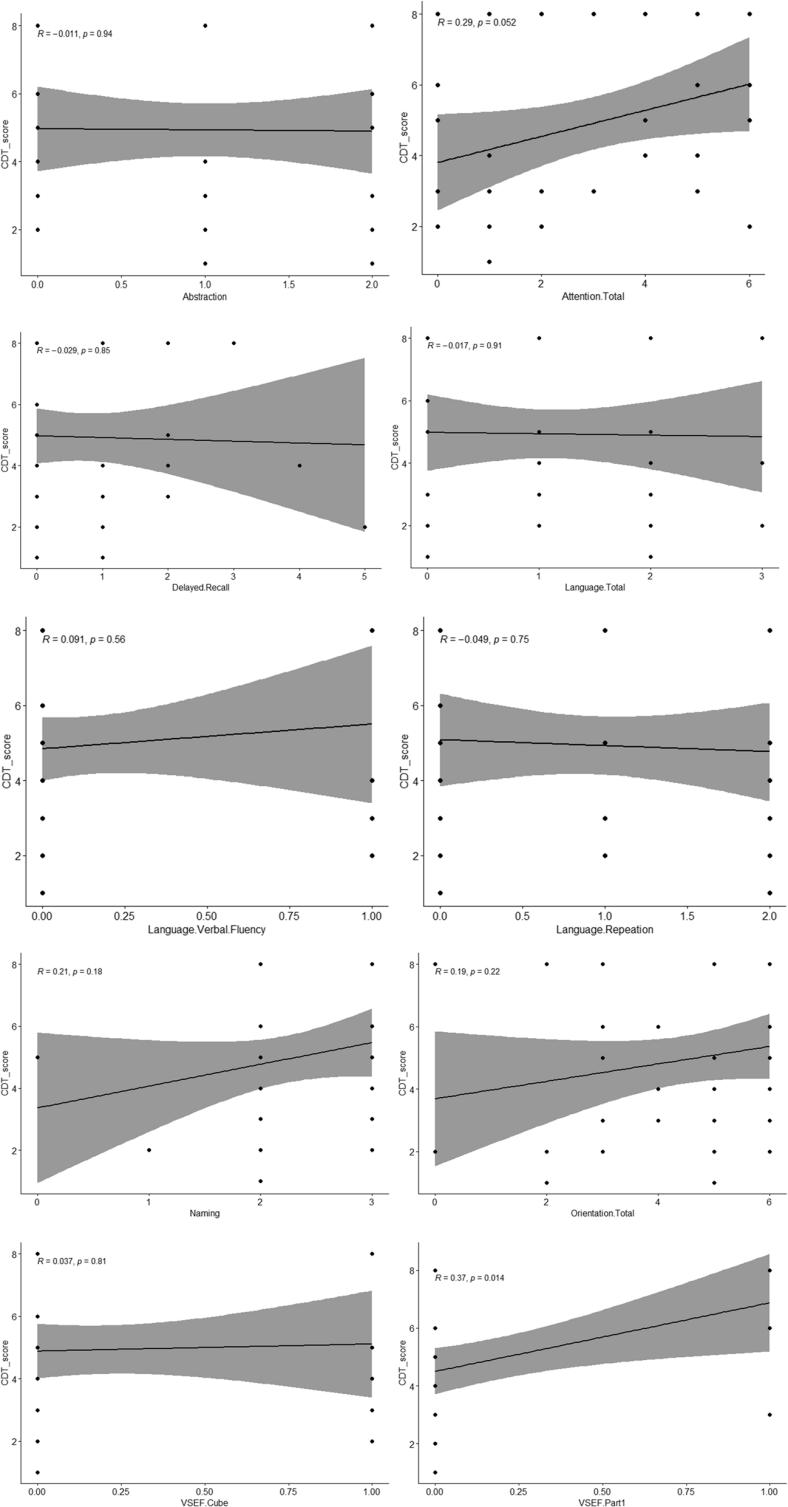
Second series of Correlations between the CDT scores and the MoCA subscales (not full CDT scores)

As it is shown in Fig. 8, the MoCA subsclaes including abstraction (*r* = −.011, *p* = .94), attention total (*r* = .29, *p* = .062), delayed recall (*r* = .029, *p* = .85), language total (*r* = −.017, *p* = .91), language verbal fluency (*r* = .091, *p* = .58), language repeatition (*r* = −.049, *p* = .75), naming (*r* = .21, *p* = .18), orientation total (*r* = .19, *p* = .22), cube (*r* = .037, *p* = .81) were not correlated with the CDT. Only visuospatial executive function had low correlation with the CDT (*r* = .37, *p* = .014).

## Discussion

Although the CDT has routinely been used to estimate the degree of impairment in dementia patients and to help diagnose patients at risk for progressive dementia [17, 18, 40–42], over the past few decades it has been used to assess other neurological disorders. Many of these non-dementing disorders include patients with lesions more focal than those seen in dementia. In this study, we showed that the CDT could not detect 30% of the patients with neurological disorders who had brain lesions. Moreover, we showed that another 29% of our patients made minimal errors on the CDT. Thus, more than half of the patients in our study were not diagnosed or properly detected using the CDT despite having a CT scan-verified brain lesion. It shows the low sensitivity of the CDT for brain lesions. In addition, the CDT was unable to predict lateralization or the location of brain lesions. Regarding the two scoring procedures we used, both qualitative and quantitative procedures performed fairly similarly. Our analyses did indicate that a small number of quantitative errors such as whether the drawn object is a clock or not are clustered separately and could be usefully added to a qualitative score.

The CDT was not able to differentiate TBI from non-TBI lesions either. This could be predictable due to the wide range of possible lesions that the patients with TBI might experience as a result of trauma. Furthermore, the analyses performed for the localization ability of the CDT did not show an unequivocal result. The parietal lobe was the only lobe with an accuracy rate of higher than 50% (i.e., 63%). However, the main strength of the test in the parietal lobe seems to be its ability to diagnose the negative cases; i.e., those without a parietal lesion. The performed non-linear classification showed a 77% specificity for parietal lesions. In other words, it could correctly identify 77% of the cases without a parietal lesion. Although not a very promising result, it has some added value in rejecting localized lesions in the parietal lobe. Regarding the frontal lobe, our classification showed a notable sensitivity for frontal lesions (67%) but a zero specificity that undermines the importance and possible clinical use of this result.

Our findings are consistent with Tranel et al’s findings [21] in which a number of their participants with a verified brain lesion did not show CDT impairment. CDT performance impairment did not accurately predict the presence of a right parietal lesion. Neither were right parietal lesions specifically related to the type of error patients made on the CDT. This is also consistent with a previous systematic review [43] which did not find any specific area of brain damage associated with clock drawing performance. It is also congruent with another study showing the lack of specificity of the CDT except for the right parietal lobe [21] and that association was only found during the acute phase of brain injury. The CDT score was also found lower in brain-injured patients with different neuroanatomical involvement, but only in an acute care setting [14]. Our findings indicate that many patients with chronic focal neurological disorders might perform relatively well on the CDT.

We showed that both qualitative and quantitative scoring systems were almost similar; the first cluster including E5, i1, j1, and k1 is likely due to impairment of executive function from prefrontal cortex lesions [38, 39]; the next cluster including c1, E1, E3 and a1 might be in result of the lateral temporal lobes dysfunctions [44], or disruption of frontostriatal circuits necessary for coordinating motor control and planning [45]; the cluster of n1, l1, E2 and E4 could be related to the time-setting instructions [35] and due to frontostriatal circuit lesions [46] or the inability to coordinate the visuospatial understanding of a clock considering the role of frontoparietal circuits [21]; The last cluster included the errors of f1, g1, b1, d1, and e1 were associated with frontostriatal and frontoparietal circuits deficits resulting in visuospatial executive dysfunctions [35]. However,in a study by Imai et al. (2022), it was suggested that the combined use of pre-drawn and free-drawn CDT method is much more sensitive to screen a wide range of brain impairments than the use of each one alone shown by ROC analysis. This method could differentiate patients with Alzheimer’s disease from MCI and healthy participants proven by significantly smaller grey matter in the bilateral temporal lobes using voxel-based morphometry [47].

Finally, we found no high meaningful correlation between the CDT and the subscales in MoCA; suggesting that the CDT is not useful in detecting cognitive impairments. It means that an impaired drawn CDT does not provide much information about the cognitive deficit of the patient except for there was an association between CDT and visuospatial impairment. However, based on our results, a normal CDT gives good news about the appropriate function of the attention of the individual. Muayqil et al. (2020) [48] also found that the MoCA clock scale (3 points system) does not have enough power to show cognitive impairments and it has to be used in companion with the MoCA to show good predictability.

## Conclusion and limitations

Our results suggest that the CDT has limited clinical validity for the assessment of patients with focal chronic brain lesions. CDT could provide more accurate information on multinetwork and multisystem lesions except for parietal lobe lesions. Furthermore, CDT is not associated with cognitive deficits in patients only with visuospatial impairments. However, our study was not without limitations. Our study was a within-patient group study and most of our participants had TBI. Future studies might consider a larger sample size with a more comprehensive cognitive assessment to assess the cognitive predictability of CDT. Furthermore, our patient’s brain lesions were assessed using brain CT scans instead of higher resolution MRI scans missing diffuse axonal injuries. Most of our patients had low education which was effective in the CDT performance.

## Supplementary Information

**Additional file 1.**

## Data Availability

The data that support the findings of this study are available from the corresponding author upon reasonable request**.**

## Abbreviations

The CDT: The Clock Drawing Test
MoCA: The Montreal Cognitive Assessment
TBI: Traumatic brain injury
Non-TBI: Non-traumatic brain injury
MCI: Mild cognitive impairment
SVM: The support vector machine
AIC: The Akaike information criterion
