# Supplementary material for: Limits on using the clock drawing test as a measure to evaluate patients with neurological disorders
Source: BMC Neurol. 2022 Dec 31;22:509. doi: 10.1186/s12883-022-03035-z (PMC9805016; doi:10.1186/s12883-022-03035-z)
Supplement: Supplementary file 1 — Additional file 1. [file 12883_2022_3035_MOESM1_ESM.docx]

| Class_ Lable  (C1: Frontal, C2: Temporal, C3: Parietal, C4: Subcortical, C5: other) | L_R  Lobe | Qualitative study of five types of error | CDT_ score |
| --- | --- | --- | --- |
| C2 |  | 0 | 10 |
| C1 | R | 0 | 10 |
| C1, C2 | R | 0 | 10 |
| C1, C2 | L | 0 | 10 |
| C3 |  | 0 | 10 |
| C2, C3 | B | 0 | 10 |
| C1 | L | 0 | 10 |
| C1, C2, C3 | R | 2 | 10 |
| C2 | R | 0 | 10 |
| C1 | L | 0 | 10 |
| C2, C3 | L | 0 | 10 |
| C1, C3 |  | 0 | 10 |
| C2 | R | 2 | 10 |
| C2 | L | 0 | 10 |
| C1 | R | 0 | 10 |
| C1 | B | 0 | 10 |
| C2 |  | 4 | 10 |
| C1 | L | 0 | 10 |
| C2 | B | 0 | 10 |
| C5 |  | 0 | 10 |
| C1 | L | 0 | 10 |
| C2 | R | 0 | 10 |
| C4 | L | 0 | 10 |
| C1 | L | 0 | 10 |
| C1, C2 | B | 0 | 10 |
| C2, C3 |  | 0 | 10 |
| C2 | R | 0 | 10 |
| C1, C2 | R | 0 | 10 |
| C3 | R | 1 | 10 |
| C1, C2 | R | 0 | 10 |

**Supplementary Table 1. The patients with full score in the CDT(Quantitative scoring)**
